# Bioelectrical impedance vector analysis and track and field jump performance across different specialties: Sex differences and electrode configuration

**DOI:** 10.14814/phy2.70035

**Published:** 2024-09-10

**Authors:** Álex Cebrián‐Ponce, Matteo Levi Micheli, Claudia Politi, Eva Bianchi, Marta Carrasco‐Marginet, Pascal Izzicupo, Gabriele Mascherini

**Affiliations:** ^1^ INEFC‐Barcelona Sports Sciences Research Group Institut Nacional d'Educació Física de Catalunya, University of Barcelona Barcelona Spain; ^2^ Department of Experimental and Clinical Medicine University of Florence Florence Italy; ^3^ Department of Medicine and Aging Sciences University “G. D'Annunzio” of Chieti‐Pescara Chieti Italy

**Keywords:** athletics, BIVA, body cell mass, phase angle, sports, track and field

## Abstract

The assessment of athletic performance using non‐invasive methods has been a significant focus in research aimed at measuring physiological parameters. This study explores the application of bioelectrical impedance vector analysis (BIVA) among track and field athletes, with a focus on sex differences, electrode configuration, and the correlation between BIVA parameters and jump performances. This cross‐sectional study involved 61 Italian track and field athletes: 31 females and 30 males (age: 21.4 ± 3.8; 21.1 ± 2.6 years; stature: 166.1 ± 6.1; 180.1 ± 5.0 cm; body mass: 57.4 ± 9.7; 72.5 ± 10.5 kg, respectively). Anthropometric measurements, bioelectrical impedance analysis, and athletic jump performance were conducted. The RXc graph, two‐sample Hotelling's T2 test for BIVA, and one‐way ANOVA for specialty comparisons were employed. Pearson and Spearman's tests evaluated the correlations between BIVA parameters and jump performance. Differences in bioimpedance values were observed between athlete groups. Lateral asymmetries were more pronounced in females. Correlations between BIVA and jump performance also varied by sex and electrode configuration, ranging from *r* = −0.072, *p* = 0.699–*r* = 0.555, *p* = 0.001 in females, and from *r* = 0.204, *p* = 0.281–*r* = 0.691, *p* = 0.001 in males. This study highlights the utility of BIVA in providing rapid and non‐invasive assessments of body composition and its relationship with jump performance, considering variations in athlete sex and electrode configuration.

## 1 INTRODUCTION

The pursuit of accurate and non‐invasive methods to evaluate athletic performance has driven exploration into measuring physiological parameters (Mendel & Cheatham, 2008). Bioelectrical impedance analysis (BIA) measures the body's resistance to externally applied alternating electrical currents, providing a straightforward method to assess athletes' body composition. Historically, BIA methods relied on two‐component models that heavily depended on hydration status to estimate body composition. These models assumed a constant fraction of total body water (TBW) in fat‐free mass (FFM), potentially leading inaccuracies in body composition estimation. However, recent advancements have introduced multi‐component models that account for variations in hydration and density, offering more precise assessments (Campa et al., 2024).

Bioelectrical impedance vector analysis (BIVA) offers an alternative approach by focusing on raw resistance (*R*) and reactance (*Xc*) values, visualized through *RXc* graphs (Piccoli et al., 1994). *R* measures current opposition, while *Xc* indicates the delay caused by cell membrane capacitance. Derived components like impedance (*Z*) and phase angle (PhA) represent vector length and direction, respectively. Vector length inversely relates to TBW, while vector direction positively reflects cellular health and membrane integrity (Campa, Gobbo, et al., 2022). BIVA (specific approach) has demonstrated a strong agreement with dual‐energy x‐ray absorptiometry (DXA) in evaluating body composition, as evidenced by significant correlations between vector length and percentage of fat mass across the entire body as well as individual body segments (Stagi et al., 2021). This strong correlation highlights BIVA's potential as a reliable method for assessing body composition. By focusing on these raw measurements, BIVA allows for a more nuanced understanding of body composition, minimizing the confounding effects of hydration.

Over the years, the method has evolved significantly, with Campa et al. (2024) refining the RXc graph ellipses to better represent population‐specific data, providing a more accurate tool for body composition assessment. Furthermore, utilizing raw data in BIA and other double indirect methods, such as anthropometry and ultrasound, offers a significant advantage by minimizing estimation errors associated with predictive equations (Campa et al., 2024). This practice enhances the reliability and precision of body composition assessments, as it circumvents the assumptions embedded in traditional predictive models, thereby providing a more accurate reflection of physiological parameters across diverse populations (Silva et al., 2023).

BIVA has found application in diverse areas including clinical settings (Norman et al., 2012; Somma et al., 2011) and sports science (Campa, Thomas, et al., 2022; Castizo‐Olier et al., 2018; Cebrián‐Ponce et al., 2021). Its use in sports has gained traction for providing rapid and non‐invasive assessments of muscle mass, body fat, and water content, crucial for the athlete's physiological profile (Campa et al., 2021). Various factors such as sex, age, and the specific physical demands of competition can influence athletes' bioelectrical impedance properties (Bongiovanni et al., 2023; Credico et al., 2021). Morphological variations among national track and field athletes across disciplines may impact bioelectrical values (Campa et al., 2020; De‐Mateo‐Silleras et al., 2023; Vučetić et al., 2008).

Each track and field discipline presents unique challenges, requiring tailored evaluation methods for events like sprints, jumps, and throws, which encompass skills such as speed, endurance, and power (Zheng & Man, 2022). Test batteries, including horizontal and vertical jump evaluations, are commonly used to assess training levels in track and field (Aoki et al., 2015). In this context, a rapid assessment of resting body composition can provide additional insights into an athlete's status. Addressing this challenge, the application of BIVA in track and field offers potential for a deeper understanding of athletes' physiological profiles as already done for athletes in other sports (Oliveira Silvino et al., 2024).

Furthermore, PhA, reflecting the relationship between *R* and *Xc*, is critical in assessing athletes' physiological status. It facilitates monitoring changes during competition (Cebrián‐Ponce et al., 2023; Izzicupo et al., 2023; Mascherini et al., 2015), identifying asymmetries (D'Hondt et al., 2021), and assessing recovery from muscle strain injuries (Nescolarde et al., 2023). PhA has also shown correlations with athletes' competitive level (Micheli et al., 2014) and endurance parameters such as VO2max (Matias et al., 2022).

This study hypothesizes that supplementing evaluations with BIVA could provide useful information to optimize sports performance in track and field. It aims to assess the applicability of BIVA across various track and field specialties and explore variations in the correlation between BIVA parameters and physical performance tests. Specifically, this study examines the differences in BIVA parameters across different track and field disciplines and athlete sexes, and how these parameters relate to jump performance.

## 2 MATERIALS AND METHODS

### 2.1 Subjects

This study adhered to the STROBE guidelines and included athletes who met the following criteria: (1) aged 18–35 years, (2) actively registered with the Italian track and field federation for the current season, (3) had a minimum of 10 years of competitive track and field experience, (4) ranked as at least tier 3 (Highly Trained/National Level) (McKay et al., 2022), (5) had no injuries or surgeries impacting sports participation within the last 3 months, and (6) were not using any medications.

The cross‐sectional study included 61 Italian track and field athletes representing diverse disciplines: 23 sprinters, 12 shot putters, 15 middle‐distance runners, and 11 jumpers. The cohort consisted of 31 female athletes (mean age ± standard deviation: 21.4 ± 3.8 years, stature = 166.1 ± 6.1 cm, body mass = 57.4 ± 9.7 kg) and 30 male athletes (mean age ± standard deviation: 21.1 ± 2.6 years, stature = 180.1 ± 5.0 cm, body mass = 72.5 ± 10.5 kg).

The tests were conducted at the Asics Firenze Marathon Stadium “Luigi Ridolfi.” Each athlete voluntarily participated in the study and received comprehensive information about the procedures and methodologies involved. Written consent was obtained from each participant in accordance with the principles of the Declaration of Helsinki 2013. Ethical approval for this study was granted by the Ethics Committee for Clinical Sport Research of Catalonia (Ethical Approval Code: 0022/CEICGC/2023) to enable retrospective data analysis. Before analysis, all physical performance data were anonymized to ensure confidentiality.

### 2.2 Procedures

**FIGURE 1:**
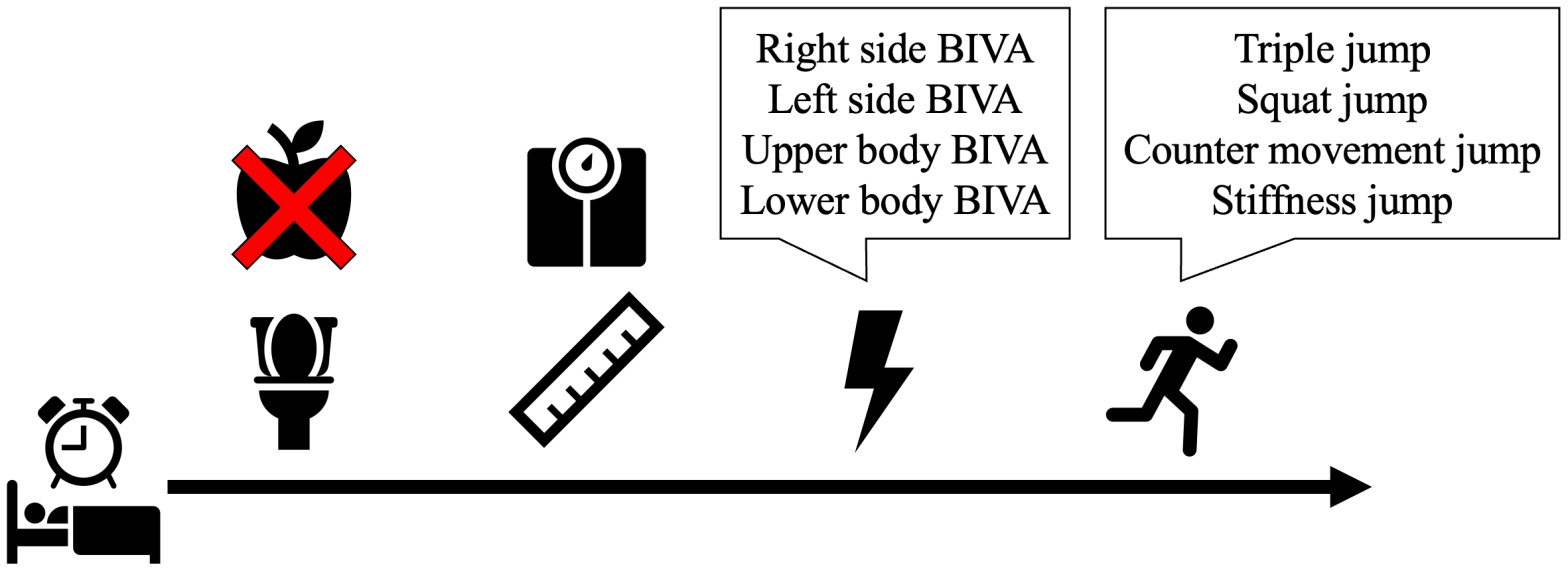
Graphical protocol of the study.

Subjects were recruited and assessed during the in‐season phase to ensure optimal physical condition. The study employed a systematic and structured assessment process, involving anthropometric, bioelectrical, and jump performance measurements as outlined in Figure 1. All assessments were conducted in the morning after participants had fasted and emptied their bowels and bladder. Additionally, athletes were instructed to abstain from consuming caffeine and alcohol, maintain their habitual dietary practice and to avoid strenuous exercise the day prior to assessments to minimize potential confounding factors.

#### 2.2.1 Anthropometric measurements

Anthropometric measurements included body mass (BM), measured to the nearest 0.1 kg, and stature, measured to the nearest 0.5 cm. BM was assessed using a calibrated mechanical column scale, the Seca 700® model, while stature was measured using a stadiometer, the Seca 220® (Seca GmbH & Co., Hamburg, Germany). Body mass index (BMI) was calculated using the standard formula: BMI = BM/stature^2^ (expressed in kg/m^2^).

#### 2.2.2 Bioelectrical measurements

**FIGURE 2:**
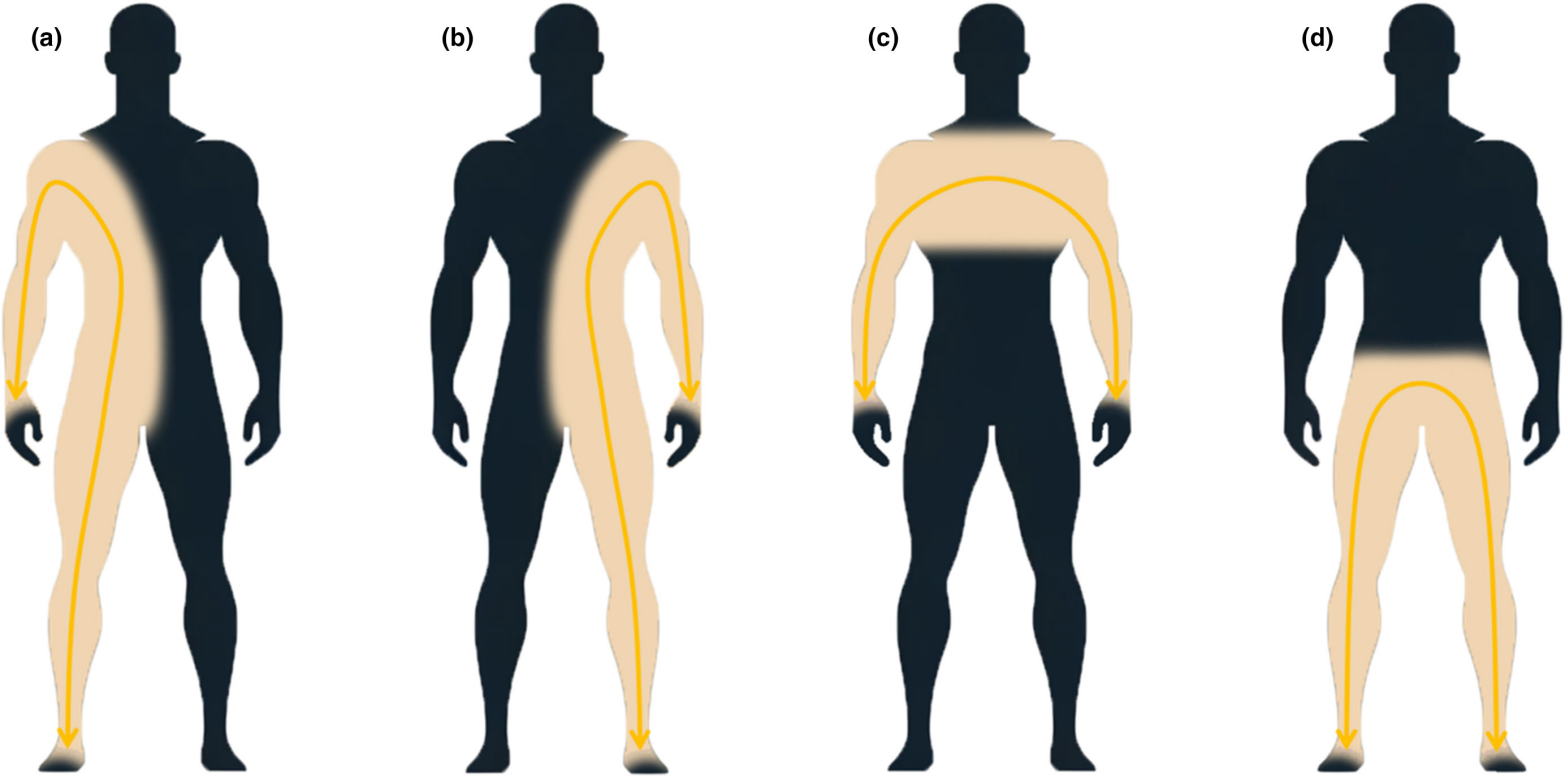
Procedures for the BIVA measurements. (a) Right‐side; (b) Left‐side; (c) Upper‐body; (d) Lower‐body.

Bioimpedance analysis was performed by a phase sensitive device (BIA 101 Anniversary AKERN srl, Florence, Italy) working with alternating sinusoidal electric current of 400 μA at an operating frequency of 50 kHz (±1%). The device was calibrated before assessment using the standard control circuit supplied by the manufacturer with a known impedance [resistance (*R*) = 380 Ω; reactance (*Xc*) = 45 Ω]. The accuracy of the device was 0.1% for *R* and 0.1% for *Xc*. For the bioelectrical impedance measurement, each participant was lying in the supine position, for at least 2 min, with a leg opening of 45° compared to the median line of the body and the upper limbs, distant 30° from the trunk. Very low intrinsic impedance (<30 Ω) disposable electrodes (Biatrodes® Akern Srl; Florence, Italy) were placed on the hands and feet bilaterally: proximal hand electrodes between the radial and ulnar styloid processes, distal hand electrodes at the center of the third proximal phalanx, proximal foot electrodes between the medial and lateral malleoli, and distal foot electrodes proximal to the second and third metatarsophalangeal joints. Measurements included the right side (hand to foot right), left side (hand to foot left), upper body (hand to hand), and lower body (foot to foot) configurations (Figure 2). Measurements were made on an isolated cot from electrical conductors and performed by the same trained investigator to minimize inter‐observer variability, ensuring data accuracy and reliability.

*Z* was calculated as (*R* ^2^ + *Xc* ^2^)^0.5^, and PhA as tan^−1^(*Xc*/*R* 180°/π). *R*, *Xc*, and *Z* were standardized for the subject's stature to remove the effect of conductor length (*R*/*H*, *Xc*/*H*, *Z*/*H*), resulting in a vector plotted on an *RXc* graph. The length of the vector on the *RXc* graph provided insights into the subjects' body fluid status, ranging from fluid overload (reduced *Z*/*H*, short vector) to fluid loss (increased *Z*/*H*, longer vector). Lateral migration of the vector indicated changes in the dielectric properties of soft tissues. Additionally, Fat Mass (FM), FFM, TBW, Extracellular Water (ECW), and Intracellular Water (ICW) were determined using the equations established by Matias et al. (2016, 2020). These parameters were calculated and plotted on RXc graphs using the right‐side configuration, following the established protocol in body composition analysis (Kyle et al., 2004).

#### 2.2.3 Jump performance tests

Before the assessment, athletes underwent a brief familiarization process to become acquainted with the procedures and to ensure their comfort with the tasks. They then completed a standard warm‐up lasting 15 min. Following this, four performance tests were conducted to evaluate the athletes' physical abilities.

The first test was the Triple Jump. Athletes began with their feet on the starting line and, when ready, executed three consecutive maximal jumps forward, alternating their supporting limbs. The distance achieved was measured from the take‐off point to the landing. Next, the Squat Jump was performed. Athletes assumed a squatting position with 90° of knee flexion, maintaining this position for approximately 2 s before jumping as high as possible. No preparatory movement was allowed, and athletes kept their hands on their hips. The jump height was recorded. The Counter Movement Jump followed, in which athletes performed a downward movement followed by full extension of the hip, knee, and ankle joints. Although they were free to determine the amplitude of the countermovement, they were instructed to keep their hands on their hips. The jump height for this test was also recorded. Lastly, the Stiffness Jump Test was conducted. Athletes performed a series of seven stiff‐legged pogos, focusing on minimizing ground contact time as much as possible. The average height of the seven jumps was recorded.

All assessments were conducted under standardized conditions to ensure accuracy and consistency in the results. Wearable inertial devices (BTS G‐Walk sensor, BTS Bioengineering, Italy) were worn during vertical jumps to capture movement data (Ridder et al., 2019). Data collected from these devices were transmitted via Bluetooth to a notebook and analyzed using BTS G‐Studio software (BTS Bioengineering, Italy). Three measurements were taken for each test, and the mean value was utilized for data analysis.

### 2.3 Statistical analysis

Data analyses were conducted using SPSS (Version 21; Chicago, IL, USA) and BIVA software (Piccoli & Pastori, 2002). Descriptive data are presented as mean ± standard deviation. An a priori power analysis was performed to determine the required sample size for this study. We anticipated a large effect size (Cohen's *d* = 0.8), and the power analysis was conducted using G*Power 3.1.9.4 software with the following parameters: for the ANOVA, an effect size of 0.8, an alpha level of 0.05, a power (1‐β) of 0.80, and four groups. The results indicated that a minimum of 24 participants per group would be necessary to detect a statistically significant difference. Regarding the *t*‐test analysis, using the same anticipated large effect size (Cohen's *d* = 0.8), the analysis showed that at least 42 participants in total would be required to achieve sufficient power for detecting the difference between two groups. Additionally, for the correlation analysis between PhA values and jump performance, a medium effect size (*r* = 0.5) was considered. The power analysis indicated that a minimum of 23 participants would be needed to detect a statistically significant correlation with sufficient power. The RXc mean graph and two‐sample Hotelling's *T* ^2^ test were utilized to assess BIA vector differences between groups and characterize them against the reference general (Campa et al., 2023) and athletic population (Marini et al., 2020). One‐way analysis of variance (ANOVA) was employed to identify potential significant differences between discipline groups, with Bonferroni post hoc tests applied. The *t*‐test was used to evaluate asymmetric differences in bioelectrical levels between the right and left sides and the upper and lower body. Correlations between PhA and jump performance tests were analyzed using Pearson and Spearman tests based on the normality of the data as determined by the Shapiro–Wilks test. Due to the small sample size when divided by groups, correlation statistics were conducted across entire sex groups. The significance level was set at *p* < 0.05.

## 3 RESULTS

**FIGURE 3:**
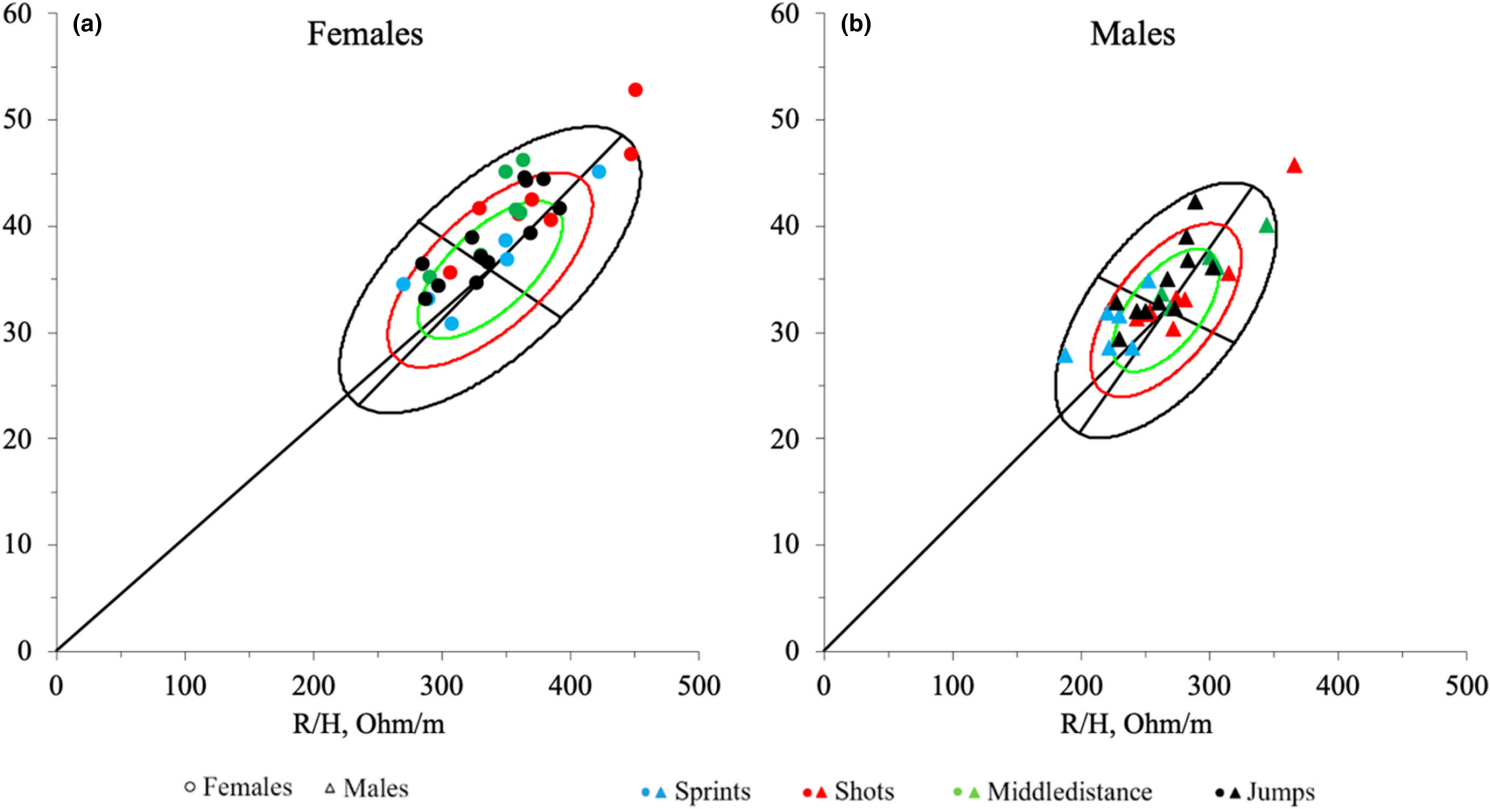
Bioelectrical impedance values of study participants, (a) female athletes and (b) male athletes, plotted on the reference ellipses of the general population.

Participants were plotted on the reference ellipses of the general population (Figure 3), updated in 2023, to provide a comprehensive comparison of bioelectrical impedance values. This approach highlights the distinct characteristics and variations in the athletes' data relative to the general population.

**FIGURE 4:**
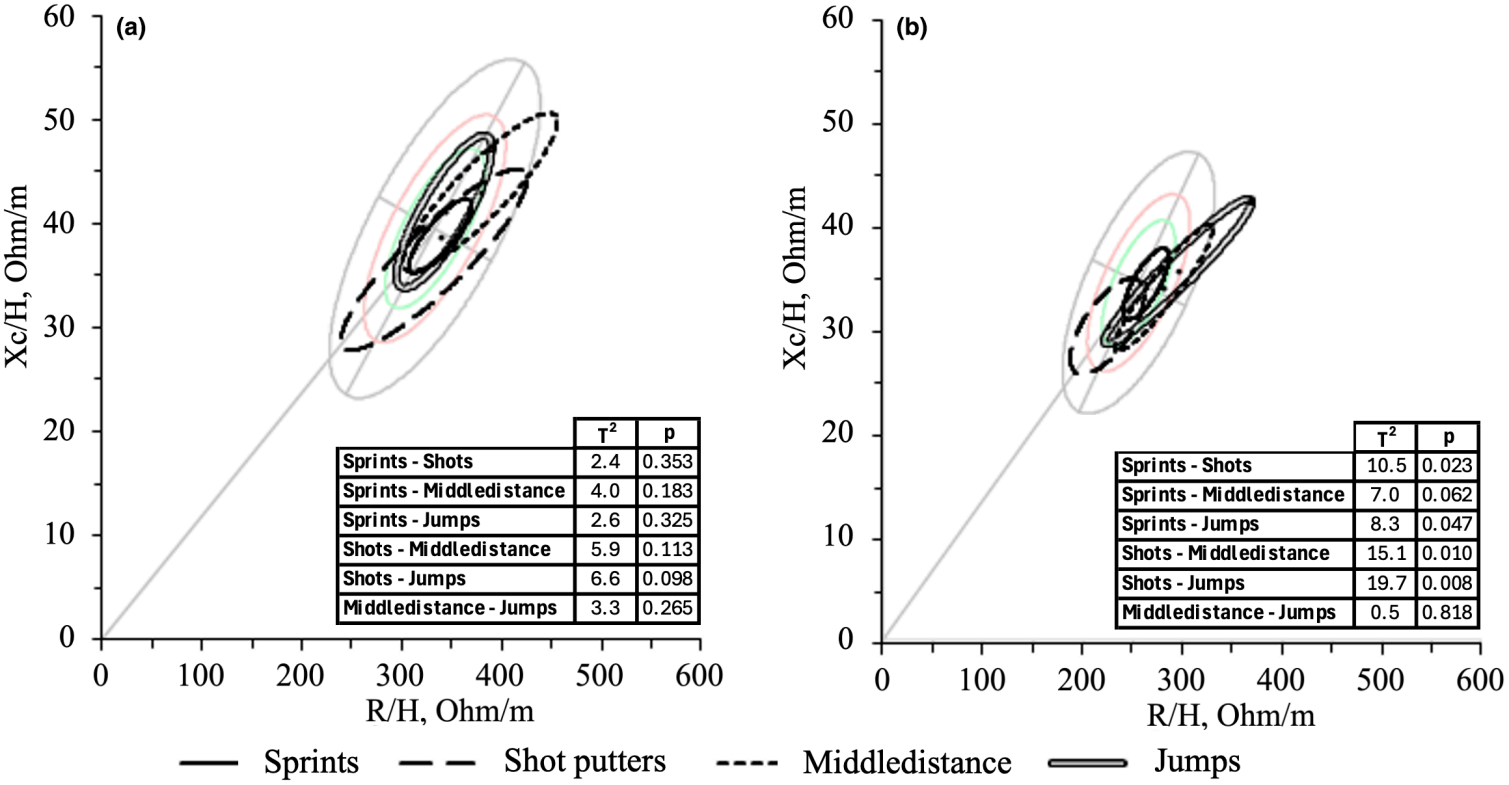
Bioelectrical differences between groups plotted in the reference athletic population. (a) Females; (b) males.

Figure 4 illustrates the placement of confidence ellipses for each group within the tolerance ellipse of the reference athletic population. Nearly all groups fall entirely within the 95% reference range. Our subjects generally show lower PhA compared to this reference population. Except for the men's jumps group, all other groups display higher *R* values than the reference values. Noteworthy are the significant differences in the BIVA complex found exclusively among males, particularly in the comparisons of sprinters, shot putters, and jumpers, as well as shot putters versus middle‐distance runners and jumpers.

Tables 1 and 2 present anthropometric, body composition parameters, bioelectrical measurements, and jump performance test results categorized by various electrode settings for track and field athletes. The sample is stratified by sex and the four track and field specialty disciplines.

**TABLE 1:** Anthropometric, bioelectrical, and jump performance measurements of the female track and field athletes.

Females						
	All	Sprinters	Shot putters	MID	Jumpers	ANOVA
	(*n*: 31)	(*n*: 12)	(*n*: 6)	(*n*: 7)	(*n*: 6)	
*Anthropometric measurements*						
BM (kg)	57.4 ± 9.7	58.3 ± 6.9	65.7 ± 14.6	47.8 ± 4.3	58.5 ± 3.7	0.005^b^, ^d^, ^f^
*H* (cm)	166.1 ± 6.1	166.3 ± 5.9	169.3 ± 6.4	161.0 ± 4.3	168.5 ± 5.0	0.045^d^, ^f^
BMI (kg/m^2^)	20.7 ± 2.5	21.1 ± 2.1	22.7 ± 3.5	18.4 ± 1.1	20.6 ± 1.4	0.012^b^, ^d^, ^f^
*Right‐body BIVA*						
*R*/*H* (Ω/m)	346.9 ± 45.0	338.2 ± 36.4	332.0 ± 54.7	378.5 ± 54.8	342.4 ± 28.0	0.205
*Xc*/*H* (Ω/m)	39.7 ± 5.0	38.8 ± 4.1	36.5 ± 5.1	43.0 ± 5.4	41.1 ± 4.3	0.086
*Z*/*H* (Ω/m)	349.2 ± 45.2	340.4 ± 36.5	334.0 ± 54.9	380.9 ± 55.0	344.9 ± 28.2	0.202
PhA (°)	6.6 ± 0.4	6.6 ± 0.4	6.3 ± 0.6	6.5 ± 0.4	6.8 ± 0.4	0.279
*Left‐body BIVA*						
*R*/*H* (Ω/m)	345.7 ± 36.8	340.6 ± 32.1	330.5 ± 56.0	362.7 ± 29.6	351.1 ± 30.0	0.430
*Xc*/*H* (Ω/m)	38.9 ± 4.8	38.2 ± 3.6	35.5 ± 4.9	40.7 ± 5.3	41.8 ± 4.5	0.083
*Z*/*H* (Ω/m)	347.9 ± 37.0	342.7 ± 32.2	332.4 ± 56.1	365.0 ± 29.9	353.6 ± 30.3	0.422
PhA (°)	6.4 ± 0.5	6.4 ± 0.4	6.2 ± 0.5	6.4 ± 0.5	6.8 ± 0.3	0.153
*Upper‐body BIVA*						
*R*/*H* (Ω/m)	364.4 ± 53.6	358.0 ± 51.6	352.0 ± 71.4	390.0 ± 57.3	360.0 ± 33.6	0.569
*Xc*/*H* (Ω/m)	38.0 ± 4.6	37.8 ± 4.0	35.1 ± 5.8	40.1 ± 5.0	38.7 ± 3.4	0.274
*Z*/*H* (Ω/m)	366.4 ± 53.7	360.0 ± 51.6	353.8 ± 71.6	392.0 ± 57.5	362.0 ± 33.7	0.567
PhA (°)	6.0 ± 0.5	6.1 ± 0.6	5.7 ± 0.5	5.9 ± 0.3	6.2 ± 0.2	0.407
*Lower‐body BIVA*						
*R*/*H* (Ω/m)	301.1 ± 41.7	292.8 ± 26.4	281.8 ± 39.2	329.1 ± 64.5	304.1 ± 25.6	0.174
*Xc*/*H* (Ω/m)	37.4 ± 5.1	36.0 ± 4.2	34.2 ± 4.2	40.2 ± 5.4	40.3 ± 5.0	0.048^d^, ^e^
*Z*/*H* (Ω/m)	303.4 ± 41.8	295.1 ± 26.4	283.9 ± 39.3	331.6 ± 64.5	306.8 ± 25.9	0.171
PhA (°)	7.1 ± 0.7	7.0 ± 0.7	7.0 ± 0.5	7.1 ± 0.8	7.5 ± 0.5	0.426
*Body composition*						
FM (kg)	13.6 ± 3.2	13.7 ± 2.9	16.3 ± 4.0	10.8 ± 1.6	13.9 ± 1.5	0.010^b^, ^d^, ^f^
FFM (kg)	43.8 ± 7.1	44.6 ± 4.8	49.3 ± 10.8	37.0 ± 4.2	44.6 ± 3.0	0.009^b^, ^d^, ^f^
TBW (L)	31.9 ± 4.9	32.4 ± 3.3	35.8 ± 7.5	27.1 ± 2.8	32.5 ± 2.1	0.008^b^, ^d^, ^f^
ECW (L)	14.1 ± 1.8	14.3 ± 1.3	15.6 ± 2.7	12.3 ± 1.1	14.2 ± 0.8	0.006^b^, ^d^, ^f^
ICW (L)	17.8 ± 3.1	18.1 ± 2.1	20.2 ± 4.8	14.8 ± 1.6	18.3 ± 1.3	0.009^b^, ^d^, ^f^
*Jump performance tests*						
TJ (m)	6.0 ± 1.0	6.0 ± 0.8	6.2 ± 0.8	5.0 ± 1.3	6.7 ± 0.3	0.013^c^, ^f^
SJ (cm)	26.5 ± 6.0	26.6 ± 7.4	27.0 ± 3.3	23.0 ± 6.3	30.0 ± 2.1	0.222
CMJ (cm)	28.8 ± 7.5	29.5 ± 9.2	29.1 ± 4.0	23.4 ± 6.7	33.6 ± 2.6	0.092
StJ (cm)	24.5 ± 6.5	23.7 ± 7.0	25.6 ± 2.3	20.0 ± 7.1	30.1 ± 3.2	0.031^e^, ^f^

Abbreviations: BIVA, bioelectrical impedance vector analysis; BM, body mass; BMI, body mass index; CMJ, counter movement jump; ECW, extracellular water; FFM, fat‐free mass; FM, fat mass; *H*, stature; ICW, intracellular water; MID, middle‐distance runners; PhA, phase angle; *R*/*H*, height‐adjusted resistance; SJ, squat jump; StJ, stiffness jump test; TBW, total body water; TJ, triple jump; *Xc*/*H*, height‐adjusted reactance; *Z*/*H*, height‐adjusted impedance.

^a^ Sprinters versus shot putters.

^b^ Sprinters versus middle‐distance.

^c^ Sprinters versus jumpers.

^d^ Shot putters versus middle‐distance.

^e^ Shot putters versus jumpers.

^f^ Middle‐distance versus jumpers.

**TABLE 2:** Anthropometric, bioelectrical, and jump performance measurements of the male track and field athletes.

Males						
	All	Sprinters	Shot putters	MID	Jumpers	ANOVA
	(*n*: 30)	(*n*: 11)	(*n*: 6)	(*n*: 8)	(*n*: 5)	
*Anthropometric measurements*						
BM (kg)	72.5 ± 10.5	70.7 ± 5.8	86.9 ± 10.2	69.9 ± 7.8	63.2 ± 6.3	0.001^a^, ^c^, ^d^, ^e^
*H* (cm)	180.1 ± 5.0	178.5 ± 5.3	180.0 ± 4.1	182.1 ± 5.2	180.6 ± 5.4	0.511
BMI (kg/m^2^)	22.3 ± 3.0	22.2 ± 1.5	26.8 ± 2.4	21.1 ± 2.0	19.3 ± 1.2	0.001^a^, ^c^, ^d^, ^e^
*Right‐body BIVA*						
*R*/*H* (Ω/m)	266.2 ± 37.5	264.0 ± 24.6	225.1 ± 21.8	281.6 ± 40.6	296.1 ± 32.6	0.003^a^, ^c^, ^d^, ^e^
*Xc*/*H* (Ω/m)	33.9 ± 4.0	34.6 ± 3.7	30.5 ± 2.7	34.2 ± 5.0	35.8 ± 3.1	0.125
*Z*/*H* (Ω/m)	268.4 ± 37.6	266.2 ± 24.8	227.2 ± 21.9	283.7 ± 40.9	298.2 ± 32.7	0.003^a^, ^c^, ^d^, ^e^
PhA (°)	7.3 ± 0.6	7.5 ± 0.5	7.8 ± 0.6	6.9 ± 0.4	6.9 ± 0.3	0.004^b^, ^c^, ^d^, ^e^
*Left‐body BIVA*						
*R*/*H* (Ω/m)	268.6 ± 36.6	265.2 ± 23.7	229.4 ± 23.8	285.7 ± 43.0	295.7 ± 22.6	0.004^a^, ^c^, ^d^, ^e^
Xc/*H* (Ω/m)	33.9 ± 3.9	34.5 ± 3.5	30.7 ± 2.9	34.3 ± 4.8	35.7 ± 2.5	0.132
*Z*/*H* (Ω/m)	270.7 ± 36.7	267.4 ± 23.9	231.5 ± 23.8	287.7 ± 43.2	297.8 ± 22.8	0.000^a^, ^c^, ^d^, ^e^
PhA (°)	7.2 ± 0.5	7.4 ± 0.5	7.6 ± 0.6	6.9 ± 0.3	6.9 ± 0.2	0.006^b^, ^c^, ^d^, ^e^
*Upper‐body BIVA*						
*R*/*H* (Ω/m)	270.4 ± 41.8	267.0 ± 22.8	223.0 ± 27.0	294.1 ± 50.0	296.8 ± 26.1	0.002^a^, ^c^, ^d^, ^e^
*Xc*/*H* (Ω/m)	31.8 ± 3.6	32.2 ± 3.0	28.1 ± 2.5	32.9 ± 4.4	33.8 ± 2.1	0.026^a^, ^d^, ^e^
*Z*/*H* (Ω/m)	272.3 ± 41.9	268.9 ± 22.8	224.7 ± 27.0	295.9 ± 50.1	298.7 ± 26.1	0.002^a^, ^c^, ^d^, ^e^
PhA (°)	6.8 ± 0.5	6.9 ± 0.5	7.2 ± 0.7	6.4 ± 0.3	6.5 ± 0.2	0.020^b^, ^d^
*Lower‐body BIVA*						
*R*/*H* (Ω/m)	241.0 ± 33.4	237.7 ± 30.9	210.5 ± 19.1	250.4 ± 32.0	269.9 ± 28.4	0.015^d^, ^e^
*Xc*/*H* (Ω/m)	32.8 ± 4.3	33.3 ± 4.1	30.4 ± 3.2	32.7 ± 5.6	34.7 ± 3.6	0.406
*Z*/*H* (Ω/m)	243.3 ± 33.5	240.1 ± 31.0	212.7 ± 19.3	252.6 ± 32.3	272.1 ± 28.5	0.016^d^, ^e^
PhA (°)	7.8 ± 0.6	8.0 ± 0.5	8.2 ± 0.6	7.4 ± 0.6	7.3 ± 0.4	0.020^b^, ^c^, ^d^, ^e^
*Body composition*						
FM (kg)	8.7 ± 3.0	8.1 ± 3.2	11.7 ± 3.0	8.5 ± 2.0	6.7 ± 1.3	0.026^a^, ^d^, ^e^
FFM (kg)	63.8 ± 8.4	62.7 ± 4.2	75.2 ± 8.0	61.4 ± 6.8	56.5 ± 5.8	0.001^a^, ^c^, ^d^, ^e^
TBW (L)	46.7 ± 5.8	45.9 ± 2.9	54.5 ± 5.5	45.1 ± 4.6	41.7 ± 3.9	0.001^a^, ^c^, ^d^, ^e^
ECW (L)	18.8 ± 2.1	18.4 ± 1.1	21.5 ± 2	18.3 ± 1.8	17.0 ± 1.5	0.001^a^, ^d^, ^e^
ICW (L)	28.0 ± 3.6	27.5 ± 1.8	33.0 ± 3.5	26.9 ± 2.8	24.8 ± 2.4	0.001^a^, ^c^, ^d^, ^e^
*Jump performance tests*						
TJ (m)	7.4 ± 0.7	7.9 ± 0.6	7.3 ± 0.8	6.7 ± 0.5	7.8 ± 0.3	0.002^b^, ^f^
SJ (cm)	36.3 ± 7.2	40.8 ± 6.1	37.8 ± 6.7	29.7 ± 6.3	35.5 ± 3.1	0.005^b^, ^d^
CMJ (cm)	39.9 ± 8.2	44.4 ± 7.6	41.1 ± 8.7	32.8 ± 6.8	39.9 ± 3.1	0.015^b^
StJ (cm)	32.8 ± 6.6	32.5 ± 7.0	34.9 ± 7.8	29.0 ± 4.4	36.9 ± 4.9	0.142

Abbreviations: BIVA, bioelectrical impedance vector analysis; BM, body mass; BMI, body mass index; CMJ, counter movement jump; ECW, extracellular water; FFM, fat‐free mass; FM, fat mass; *H*, stature; ICW, intracellular water; MID, middle‐distance runners; PhA, phase angle; *R*/*H*, height‐adjusted resistance; SJ, squat jump; StJ, stiffness jump test; TBW, total body water; TJ, triple jump; *Xc*/*H*, height‐adjusted reactance; *Z*/*H*, height‐adjusted impedance.

^a^ Sprinters versus shot putters.

^b^ Sprinters versus middle‐distance.

^c^ Sprinters versus jumpers.

^d^ Shot putters versus middle‐distance.

^e^ Shot putters versus jumpers.

^f^ Middle‐distance versus jumpers.

Differences were detected among various track and field disciplines concerning anthropometric measurements (BM and BMI) and body composition metrics (FM, FFM, TBW, ECW, and ICW). The only groups exhibiting discrepancies across all these metrics in both genders were the shot putters and middle‐distance runners.

Regarding bioelectrical measurements, no notable differences were observed within female cohorts across different electrode configurations, except for reactance per height (*Xc*/*H*) between shot putters versus middle‐distance runners and jumpers at the lower limbs. Conversely, male athletes displayed numerous disparities between track and field specialties. Specifically, PhA in the male group showed significant differences between middle‐distance runners versus sprinters and shot putters, regardless of electrode configuration. Jumpers significantly differed from middle‐distance runners and shot putters in PhA in all groups except for upper‐body BIVA.

Regarding jump performance assessments, distinctions between groups were evident in both genders solely in the triple jump. However, male athletes demonstrated discrepancies in the squat jump and countermovement jump, while female athletes showcased variations in the stiffness jump test.

Table 3 illustrates differences in asymmetry among track and field specialties, categorized by sex. Female athletes, especially jumpers, showed noticeable differences in asymmetry between the right and left sides. However, significant differences between the right and left sides within each category were absent among male athletes, except for resistance per height (*R*/*H*) and PhA in the overall male group. Furthermore, more differences were observed in both sexes when bioelectrical data between the upper and lower bodies were compared.

**TABLE 3:** Bioelectrical variances between right–left BIVA and upper–lower BIVA.

		Right‐left				Upper‐lower			
		Females		Males		Females		Males	
		*t*	Sig.	*t*	Sig.	*t*	Sig.	*t*	Sig.
All	*R*/*H* (Ω/m)	0.39	0.698	−1.52	0.140	7.24	**0.001**	6.46	**0.001**
	*Xc*/*H* (Ω/m)	2.71	**0.011**	0.00	1.000	0.95	0.348	−1.88	0.071
	*Z*/*H* (Ω/m)	0.42	0.680	−1.49	0.146	7.22	**0.001**	6.37	**0.001**
	PhA (°)	2.82	**0.008**	2.16	**0.039**	−7.75	**0.001**	−11.27	**0.001**
Sprinters	*R*/*H* (Ω/m)	−0.69	0.505	−0.63	0.545	4.70	**0.001**	3.71	**0.004**
	*Xc*/*H* (Ω/m)	2.43	**0.034**	0.72	0.491	1.89	0.086	−1.38	0.197
	*Z*/*H* (Ω/m)	−0.67	0.518	−0.60	0.562	4.71	**0.001**	3.64	**0.005**
	PhA (°)	3.59	**0.004**	1.70	0.120	−3.10	**0.010**	−8.16	**0.001**
Shot putters	*R*/*H* (Ω/m)	0.42	0.695	−1.63	0.164	4.00	**0.010**	1.75	0.141
	*Xc*/*H* (Ω/m)	2.41	0.061	−1.11	0.318	0.86	0.428	−2.77	**0.040**
	*Z*/*H* (Ω/m)	0.45	0.671	−1.63	0.164	3.98	**0.011**	1.68	0.153
	PhA (°)	2.08	0.093	1.28	0.256	−10.18	**0.001**	−6.64	**0.001**
MID	*R*/*H* (Ω/m)	1.44	0.199	−1.07	0.322	2.13	0.077	4.27	**0.004**
	*Xc*/*H* (Ω/m)	2.56	**0.043**	−0.56	0.595	−0.16	0.876	0.20	0.851
	*Z*/*H* (Ω/m)	1.46	0.195	−1.06	0.326	2.12	0.078	4.26	**0.004**
	PhA (°)	0.71	0.504	0.84	0.430	−3.21	**0.018**	−4.18	**0.004**
Jumpers	*R*/*H* (Ω/m)	−2.97	**0.031**	0.08	0.940	7.77	**0.001**	6.67	**0.003**
	*Xc*/*H* (Ω/m)	−2.93	**0.033**	0.38	0.725	−1.63	0.164	−0.78	0.481
	*Z*/*H* (Ω/m)	−3.03	**0.029**	0.08	0.940	7.69	**0.001**	6.50	**0.003**
	PhA (°)	0.44	0.679	0.00	1.000	−8.33	**0.001**	−3.98	**0.016**

*Note*: Significant differences (*p* < 0.05) are marked in bold.

Abbreviations: MID, middle‐distance runners; PhA, phase angle; *R*/*H*, height‐adjusted resistance; *Xc*/*H*, height‐adjusted reactance; *Z*/*H*, height‐adjusted impedance.

**FIGURE 5:**
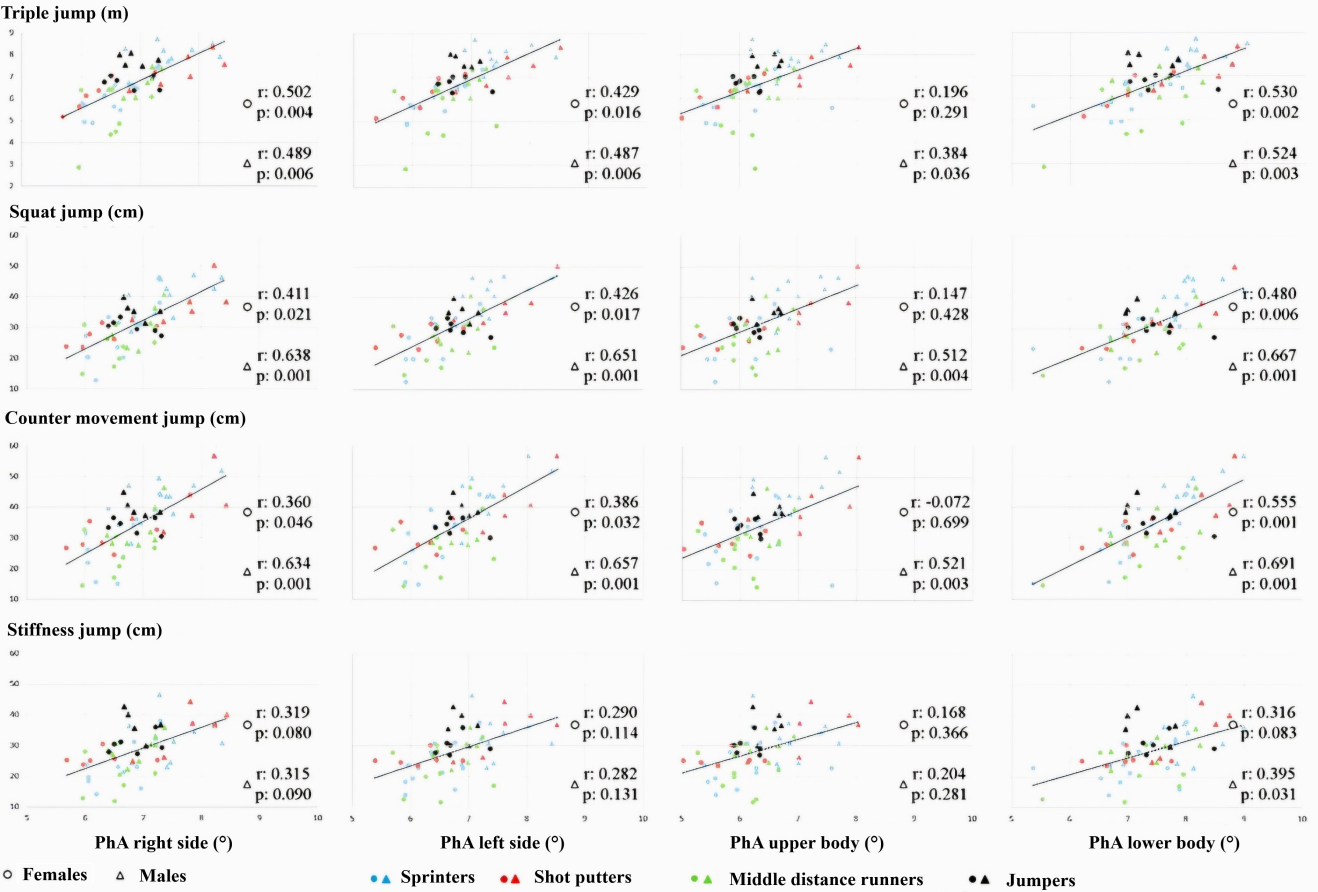
Graphic representation grid depicting correlations between phase angle across various electrode configurations and jump performance tests. PhA, phase angle (°).

Figure 5 displays a grid of graphical correlations between the PhA of each electrode configuration (right side, left side, upper body, and lower body) and the performance results (triple jump, squat jump, counter‐movement jump, and stiffness jump). Different configurations exhibit some variations. The highest correlations are observed in the squat (ranging from 0.426 to 0.667) and counter‐movement jumps (ranging from 0.360 to 0.691), with the only exception being the lack of correlation in the upper body BIVA of female athletes. In the triple jump, correlations range from 0.384 to 0.530, except for the women's group on the upper body. Regarding the stiffness jump test, the only significant correlation is found in the males' group on the lower body (*r*: 0.395, *p* = 0.031).

The male group shows a greater correlation between PhA and jump performance tests, with 13 significant correlations out of 16. The group of female athletes shows 9 out of 16. The positioning of the electrodes shows six out of eight significances for the right side, six for the left side, three for the upper body, and seven for the lower body.

These relationship results will be analyzed considering three key aspects: (1) horizontal and vertical jump tasks, (2) the athlete's sex, and (3) different body part analyzed (electrode configuration patterns). A higher degree of correlation is observed for squat and countermovement jumps in males, particularly evident with lower‐body BIVA (*r* = 0.667, *p* < 0.001; *r* = 0.691, *p* < 0.001). Similarly, for female athletes, the highest degree of correlation is seen in the analysis of the lower‐body PhA, specifically with the triple jump and countermovement jump tests (*r* = 0.530, *p* = 0.002; 0.555, *p* < 0.001).

## 4 DISCUSSION

This investigation has provided insights into the applicability of BIVA across various specialties within track and field sports. Our study focused on correlating BIVA parameters with jump performance tests associated with each track and field event to optimize BIVA utilization and enhance sensitivity with each test. Anthropometric and body composition parameters unveiled significant differences between track and field specialties, particularly in BM, BMI, FM, FFM, and fluid distribution among male and female athletes, consistent with previous findings (O'Connor et al., 2007). The body composition values from our study and the differences observed between specialties align with those reported in previous studies on NCAA Division I collegiate track and field athletes who used DXA (Hirsch et al., 2016). However, they differ from the findings of Stone et al. (2024), who conducted a study on an American university track and field team using bioelectrical impedance, where no distinction was made between the different specialties.

### 4.1 BIVA analysis of track and field specialties

Figure 4 presents a comprehensive BIVA analysis comparing different track and field specialties. The majority of the analyzed groups fell within the 95% confidence interval, indicating a general consistency in body composition relative to the reference athletic population. There was a trend towards lower PhA values in the study subjects compared to the reference population, suggesting potential variations in the quality of body cell mass (Kyle et al., 2004). Consistent with Campa, Gobbo, et al. (2022), PhA differed across sport modalities, with male athletes exhibiting a higher PhA than female athletes within each sport modality. Male and female sprinters were positioned almost within the 50% tolerance ellipse. In contrast, male and female middle‐distance runners were in the upper right quadrant, indicating relatively less muscle mass. A distinct pattern emerged between the sexes for jumpers, with males showing less muscle mass and shot putters with higher water content in males and heterogeneity in females.

Significant differences in the BIVA complex were exclusively observed among different specialties in male track and field athletes, indicating potential sex‐specific variations in bioelectrical impedance patterns that warrant further investigation. These observations underscore the importance of considering sex‐specific variations and sport‐specific demands when interpreting bioelectrical impedance measurements in athletic populations. Although anthropometric and body composition parameters showed significant differences between track and field specialties, whole‐body BIVA analysis appeared to reshape these differences in females.

### 4.2 Bioelectrical impedance patterns and sex differences

Female athletes exhibited the only significant difference in *Xc*/*H* at the lower limbs between shot putters and middle‐distance runners/jumpers (Table 1). Conversely, in male athletes, this was the only analysis that showed no differences (Table 2). This finding aligns with previous studies describing differences in lower limbs among athletes from other sports (Mascherini et al., 2017). These differences are likely due to BIVA current flow passing through lean mass, which is more represented in males than in females. The observation of significant differences in BIVA suggests the influence of specific physical demands of each specialty on athletes' body composition. Since this is the first study using the BIVA methodology in track and field sports, further investigations are necessary to expand the study sample and possibly include international‐level athletes.

### 4.3 Asymmetry in track and field athletes

BIVA comprehensively assesses body composition, fluid distribution, and body cell mass, revealing potential asymmetries between the body's sides (Stagi et al., 2023). While some degree of asymmetry is normal and can even enhance performance in specific sports (Fox et al., 2023), excessive or imbalanced asymmetry may increase the risk of injury and hinder overall performance (Michalski et al., 2017). Sex‐specific differences in asymmetry among various track and field specialties are illustrated in Table 3. Men display greater symmetry across all groups, although there is an asymmetry between the right and left sides in PhA observed in the entire male sample, with PhA being higher on the right side for both sexes. Additionally, the *R*/*H* ratio is higher on the right side in women, while in men, it is higher on the left side. The most noticeable asymmetry between the right and left sides is seen in the women's jumping group, with significantly lower values on the right side in *R*/*H*, *Xc*/*H*, and *Z*/*H* but not in PhA. Regarding upper‐lower differences, disparities are evident in all groups, as expected due to the varying impedance properties of body segments (Stagi et al., 2021). Asymmetries in the upper limbs may affect throwing or striking performance (Bauer et al., 2021). In contrast, the lower limbs can impact an athlete's ability to generate power and stability during explosive movements like sprinting or jumping (Fox et al., 2023). For instance, a study revealed PhA side‐to‐side asymmetry between tennis players' dominant and non‐dominant upper limbs, attributed to differences in lean mass at the upper limb (D'Hondt et al., 2021).

### 4.4 Correlations between BIVA and jump performance tests

The correlations between BIVA and jump performance tests aim to delineate differences among same‐sex athletes in various specialties. PhA was used as a parameter linked to body composition to evaluate the degree of correlation with jump performance since its reliability in this analysis in different types of study populations, both sporting and non‐sporting (Cirillo et al., 2023; Custódio Martins et al., 2022). From a physiological point of view, the observed relationship between lower body PhA and jump performance is expected, as the lower limbs are primarily responsible for executing jumps. Additionally, both PhA and jump height tend to be greater in male athletes. However, the stiffness test exhibits little relationship with BIVA, likely because the task mainly involves a small body part, such as the calf muscles, which may not significantly contribute to the overall bioimpedance measurements.

Male track and field athletes demonstrate more correlations than females (13 vs. 9), as the analyzed performances are closely linked to muscular strength and, consequently, lean mass, which predominates in males (Barbieri et al., 2017). Conversely, speed and strength performance in female athletes appears to be more associated with FM (Abe et al., 2020). Future studies could explore endurance performance and examine its relationship with FM to elucidate these findings further. As expected, the electrode configuration pattern reveals a higher frequency of correlations between the lower‐body, given the predominant involvement of the lower limbs in jumping performances (Marra et al., 2016). However, it is worth noting that the differences between the right and left sides must be more substantial to warrant consideration. Interestingly, PhA of the upper body exhibits no significant relationship with squat and countermovement jumps in females, contrasting with males, possibly due to the higher lean mass in the upper body of male athletes compared to females.

### 4.5 Jump performance and specialty differences

The analysis did not explore differences in jump performance tests between sexes, given the well‐documented gap of around 10%–12% in the literature (Ospina Betancurt et al., 2018). Instead, the focus was on delineating differences between specialties among athletes of the same sex. Notably, a more pronounced influence of track and field specialties was observed in female athletes, with jumpers consistently achieving higher or longer distances (Tables 1 and 2). Conversely, among males, sprinters demonstrated comparable results to jumpers, possibly due to the strong lower limb strength production required during sprinting and jumping tasks and the relationship between the horizontal direction of force production during sprinting and the triple jump (Dietze‐Hermosa et al., 2021).

### 4.6 Limitations and future perspectives

While our study yields valuable insights, some limitations must be acknowledged. Firstly, the cross‐sectional design and small sample size limit the generalizability of the findings. Power calculations showed that 24 participants per group were needed to detect significant differences. For correlations, a minimum of 23 participants was required. Larger studies are needed to enhance generalizability. However, our sample size aligns with previous track and field studies. Secondly, our study did not assess the differences in jumping performance between the right and left lower limbs in relation to the asymmetry of bioelectrical impedance parameters. This analysis could have offered additional insights into the physical characteristics of the athletes. Nonetheless, this can be considered as a potential direction for future research. Thirdly, due to time constraints during fieldwork, we were unable to synchronize testing with the same menstrual cycle phase for all female athletes. We acknowledge this limitation as a potential source of variability in bioelectrical impedance measurements, as fluctuations during the menstrual cycle could influence the results. Finally, the bioelectrical data was evaluated using a sampling rate of 50 kHz. Therefore, the results of this study cannot be extended to other impedance device solutions.

Further research with larger and more diverse cohorts is needed to corroborate our findings, taking into account different phases of the menstrual cycle.

### 4.7 Implications for training and performance optimization

Despite its limitations, our study offers promising implications for training and performance optimization:

- Integration of BIVA: Bioelectrical Impedance Vector Analysis (BIVA) has the potential to personalize training programs based on responses to performance tests across various track and field specialties. While our study focused on jumping performance, it is reasonable to hypothesize that BIVA could also be effective for evaluating performance in upper limb sports.
- Application to training and sports science: coaches and sports scientists could utilize BIVA data to design targeted interventions that address asymmetries in the upper/lower body and left/right sides, considering the specific demands of different sports (e.g., throwing vs. running) or for managing return‐to‐sport protocols following injury.
- Contribution to sports science: this research enhances our understanding of bioelectrical characteristics across different track and field specialties, thereby laying the groundwork for future studies on the relationship between physical performance and raw bioelectrical parameters.

## 5 CONCLUSIONS

In summary, our study contributes to the evolving field of sports science by evaluating the applicability of BIVA in track and field sports. Our findings demonstrate that BIVA offers quick and non‐invasive assessments of FFM, body fat, and water content in track and field athletes. The correlations observed between BIVA parameters and jump performance tests, along with identifying specialty‐specific differences based on electrode configuration and athlete sex, highlight the potential of BIVA in enhancing athletic performance evaluation. Future research incorporating larger sample sizes and longitudinal designs must fully unlock the nuanced relationship between bioelectrical impedance patterns, physiological adaptations, and performance outcomes in track and field athletes.

## AUTHOR CONTRIBUTIONS

Conceptualization: Matteo Levi Micheli and Gabriele Mascherini; Methodology: Álex Cebrián‐Ponce, Pascal Izzicupo, Matteo Levi Micheli; Formal analysis: Álex Cebrián‐Ponce, Pascal Izzicupo; Investigation: Claudia Politi, Eva Bianchi; Writing—original draft preparation: Álex Cebrián‐Ponce, Gabriele Mascherini, Pascal Izzicupo; Writing—review and editing the draft: Marta Carrasco‐Marginet; Resources: Gabriele Mascherini and Matteo Levi Micheli; Supervision: Gabriele Mascherini and Matteo Levi Micheli; All authors read and approved the final manuscript.

## FUNDING INFORMATION

This research received no external funding.

## CONFLICT OF INTEREST STATEMENT

The authors declare no conflict of interest.

## ETHICS STATEMENT

The study was conducted in accordance with the Declaration of Helsinki, and approved by the Ethics Committee for Clinical Sport Research of Catalonia (Ethical Approval Code: 0022/CEICGC/2023).

## INFORMED CONSENT STATEMENT

Informed consent was obtained from all subjects involved in the study.

## Data Availability

The data supporting this study's findings are available in Mascherini, Gabriele; Levi Micheli, Matteo (2024), “Track and Field & BIVA,” Mendeley Data, V1, doi: 10.17632/ttnpgykg39.1.
